# The time of emergence of Arctic warming, wetting and sea ice melting

**DOI:** 10.1038/s41598-025-96607-1

**Published:** 2025-04-12

**Authors:** Nicoleta Tsakali, Marlen Kolbe, Richard Bintanja, Nomikos Skyllas

**Affiliations:** 1 Royal Netherlands Meteorological Institute (KNMI), De Bilt, the Netherlands; 2 Energy and Sustainability Research Institute Groningen (ESRIG), University of Groningen, Groningen, the Netherlands

**Keywords:** Climate sciences, Climate and Earth system modelling, Projection and prediction

## Abstract

In the rapidly warming and wetting Arctic, the time of emergence (ToE) of a new climate state occurs when trends of climate indicators are large enough to surpass the strong natural climate fluctuations in the Arctic. Thus far, uncertainties in climate model projections, variability and methods have yielded diverging estimates of Arctic ToE. Here we use a robust method and future projections of multiple state-of-the-art climate models to show that, generally, sea ice thickness (2036–2051) and surface air temperature (2033–2050) emerge first, followed by sea ice cover (2039–2074), and precipitation/rainfall (after 2077). Autumn generally exhibits the earliest ToE-values due to rapid sea ice retreat. The earliest ToE for temperature and sea ice thickness occurs in the Central Arctic, whereas sea ice cover and rainfall first emerge in the Barents Sea region. Most regions of the Arctic are close to a new climate state (for temperature and sea ice), with wide-ranging and possibly irreversible consequences for vulnerable Arctic ecosystems and human activities.

**Supplementary Information:**

The online version contains supplementary material available at 10.1038/s41598-025-96607-1.

## Introduction

In view of recent global increases in the occurrence of climate change-related natural disasters^1–3^, there is enhanced interest in determining whether extreme climate conditions are a regular feature of the current climate, or if they pose evidence of a new climate state having emerged. One of the most vulnerable regions to climate change is the Arctic, which is warming two to four times faster than the global average over the last decades^4–6^, while also getting considerably wetter^7–10^. Understanding when the effects of anthropogenic climate change in the Arctic have/will become distinguishable from natural climate fluctuations is crucial for policymakers to develop effective mitigation and adaptation strategies, and to identify and quantify (potentially irreversible) impacts on vulnerable Arctic ecosystems. A useful metric to establish when the forced climate change trend emerges from the ‘background noise’, or natural climate variability, is the time of emergence (ToE).

Thus far, the few studies that addressed Arctic ToE have provided inconclusive and diverging results, mainly because the methods to evaluate ToE and the climate variables/metrics that have been assessed differ greatly^11–16^. Previous estimates of ToE for Arctic Amplification^12^, surface air temperature^16^, shortwave radiation^11^, sea ice extent (SIE)^16^, the freshwater budget^15^, or solar absorption^14^ focused on only one or a few variables and concluded that the forced trend has either already emerged or will likely emerge within the current decade^13,16^. For rainfall, only the ToEs for the first, last, and duration of Arctic rain days were evaluated and found to occur later this century^16^. Studies that evaluated Arctic ToE were sometimes based on spatial and/or temporal averaging (which reduces variability and hence results in an earlier ToE, especially in sea ice, see Supplementary Information Figs. 1 and 2), as such, these have mostly attributed emergence to processes associated with Arctic sea ice decline^12,16,17^. For instance, a recent study suggested that the Arctic climate has already emerged decades ago in terms of both the seasonal minimum and maximum of SIE^16^, but these estimates are based on Arctic average SIE, which due to spatial averaging of variability yield an unrepresentative early ToE. To date, crucial Arctic variables such as sea ice thickness have not been analysed in terms of their first emergence. Precipitation/rainfall exhibit strongly increasing trends, and also considerable variability, but so far ToE has been evaluated only for specific precipitation indices and metrics based on only two climate models^16^. As a result, ToE-estimates are often hard to intercompare because of differing climate variable indices and metrics, as well as differing methodology such as, most crucially, spatial and temporal averaging. Evidently, there is an urgent need for a complete, reliable and internally consistent estimate of the first emergence of the Arctic climate – including the associated uncertainties – based on a broad range of state-of-the-art climate models and five climate variables representative of the Arctic climate, including their geographical and seasonal variations.

## Results

### Arctic mean time of emergence

**Fig. 1:**
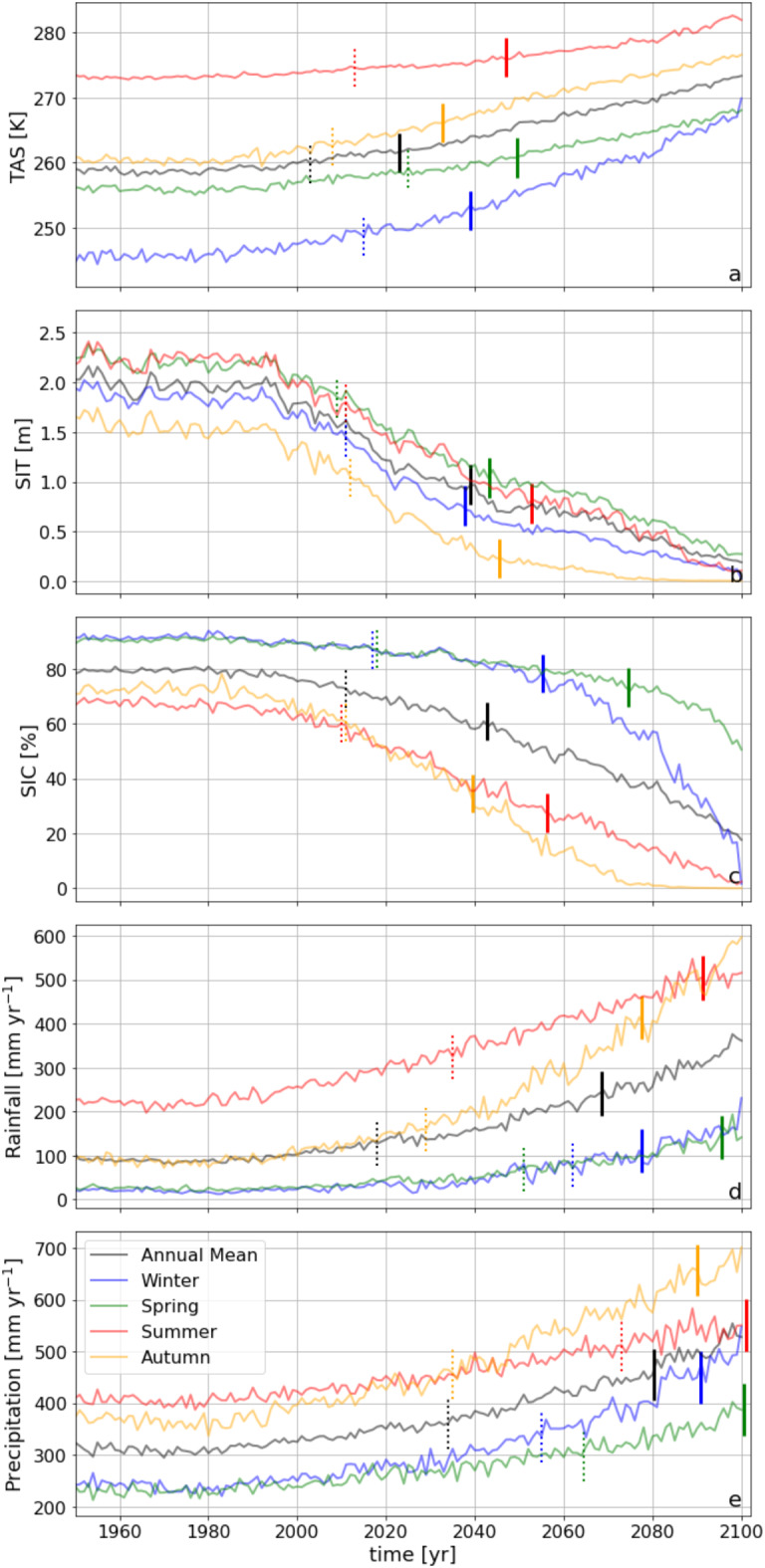
Seasonal and annual Arctic mean timeseries of (**a**) surface temperature, (**b**) sea ice thickness, (**c**) sea ice cover, (**d**) rainfall, and (**e**) total precipitation over 150 years (historical and SSP5.85 CMIP6 simulations, median of CMIP6 models). Vertical lines depict the Time of Emergence calculated using the Arctic mean (dotted lines) and per grid point method (comBPnn, continuous lines).

Here we use a detailed methodology (see Methods) to robustly determine Arctic ToE from state-of-the-art climate model simulations using 15 models in standardised scenario simulations (CMIP6, historical and SSP5-8.5)^18^ to show that, averaged over the Arctic (70°N-90°N), the seasonal emergence of surface air temperature (TAS, 2033–2050) and sea ice thickness (SIT, 2036–2051) will precede sea ice cover (SIC, around 2039–2074), followed by rainfall and precipitation (2077–2096, 2090-after the end of the century, respectively) (Fig. 1). In contrast to seasonal means, annual means average out seasonal variability; hence we find that annual mean ToE-estimates precede seasonal values by 14 ± 5 years (Fig. 1). Strong future trends result in an earlier ToE, but a large variability – and/or an increase in variability – will delay ToE (Supplementary Information Fig. 6). The later emergence of rainfall/precipitation in spite of strong future trends can be attributed to comparatively large past and present variability as well as to further future increases in variability^19^. Until now, estimates of ToE for sea ice changes were based on Arctic mean/total sea ice concentration, thus averaging out spatial variability, which likely resulted in too early ToE-values (up to 56 years compared to our best estimates, see Supplementary Information Figs. 1 and 2). Crucially, we find that evaluating the sea ice cover emergence per grid point considerably delays ToE to just after that of temperature (Fig. 1). These results suggest that, in contrast to earlier studies, the mean Arctic climate, despite exhibiting trends that are among the largest in the world^4–6^ through local amplifying feedbacks, has overall not yet moved significantly outside the current state (although some regions and variables have already emerged). However, ToE values exhibit considerable intermodel differences (Supplementary Information Fig. 3), indicative of diverging model-specific simulated Arctic trends and variability – interannual and/or decadal – as well as the associated governing processes/feedbacks^20,21^. This clearly indicates that analyses of ToE should be based on as many models as possible to accurately quantify the associated uncertainties.

## Regional and seasonal variations in ToE – temperature and sea ice

**Fig. 2:**
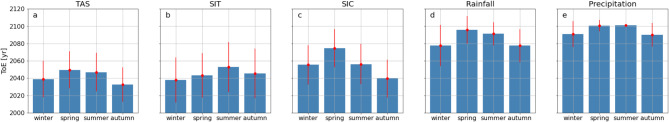
Multi-model estimates of the seasonal and Arctic means Time of Emergence (ToE) for (**a**) surface temperature, (**b**) sea ice thickness, (**c**) sea ice cover, (**d**) rainfall and (**e**) total precipitation. The values are the median ToE (calculated using our per grid point method: comBPnn) of all CMIP6 models for the entire Arctic. The red vertical lines represent the model uncertainty (standard deviation of model ensemble).

**Fig. 3:**
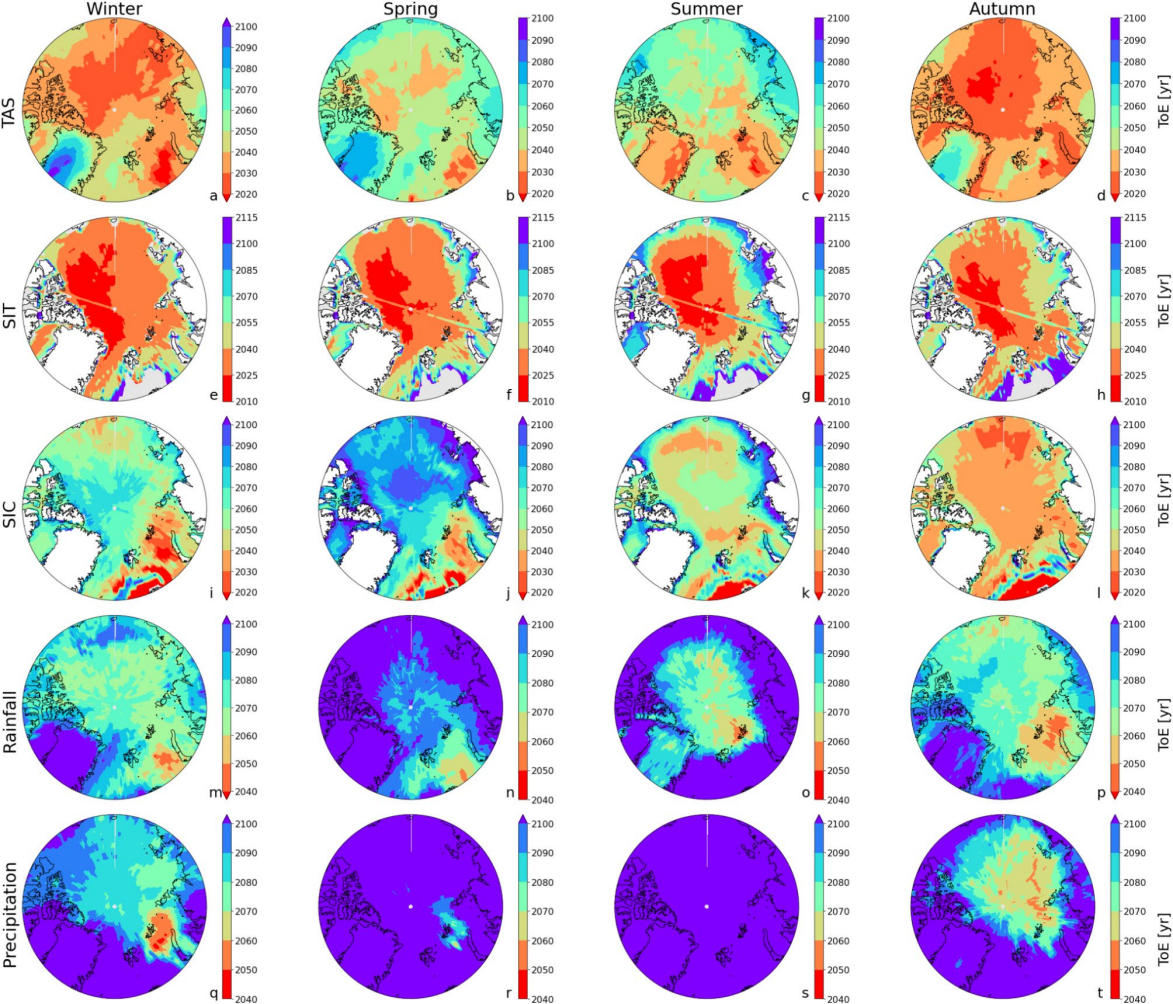
Geographical distribution of the seasonal Time of Emergence (ToE) for surface temperature (first row), sea ice thickness (second row), sea ice cover (third row), rainfall (fourth row) and total precipitation (fifth row). The grid cell values are the median ToE of the CMIP6 models calculated using the per grid point method (comBPnn), for winter (DJF, first column), spring (MAM, second column), summer (JJA, third column), autumn (SON, fourth column). For each model, grid points failing to produce a ToE-value during the 21st century (≤ 2100) are assigned a ToE-value of 2101. Purple colour depicts the grid cells where the median of the CMIP6 models is 2101, while white colour depicts land without SIT and SIC. Note the different colour scales per variable.

TAS, SIC, and SIT are expected to transition to a new state across the Arctic by the end of this century, with changes occurring in all seasons (Fig. 1a-c). The earliest transitions are projected for autumn TAS, starting as soon as 2033 (Fig. 1a). SIT generally transitions around the same time as TAS, except for autumn, where TAS leads SIT by 13 years (Figs. 1a-b and 2a-b). Notably, some variables and regions have already emerged, such as SIT in the Central Arctic (Fig. 3e-h), while others, like winter TAS over Greenland (Fig. 3a) and spring SIC in most regions (Fig. 3j), are not expected to emerge until after 2100.

The presence of sea ice influences the emergence of TAS particularly during warmer seasons, such as summer and autumn, when temperatures reach the melting point of ice. Once this point is reached, further warming is limited until the ice fully melts, resulting in TAS and SIC transitions occurring almost simultaneously during these months (Fig. 2a, c). However, in colder seasons, such as winter and spring, TAS remains well below the melting point and can thus increase freely. As a result, TAS transitions occur up to 24 years earlier than SIC in spring (Fig. 2a, c). This pattern is particularly pronounced in the Central Arctic, where TAS is expected to transition before 2050 in spring (Fig. 3b), while SIC transitions will not occur until after 2070 or, in many areas, not even before the end of this century (Fig. 3j). In contrast, in regions with thin sea ice, such as the Barents Sea, the relationship between TAS and SIC is more synchronized, with both transitioning relatively early (Fig. 3b, j) due to the already thin ice which suggests that surface temperatures have already reached the melting point. When analyzing individual models, we find that those with an earlier ToE in temperature also tend to exhibit an earlier ToE in SIC (Supplementary Information Fig. 9). This approximately linear relationship is most pronounced in summer (*R* = 0.96), likely due to relatively high temperatures efficiently melting comparatively thin sea ice.

**Fig. 4:**
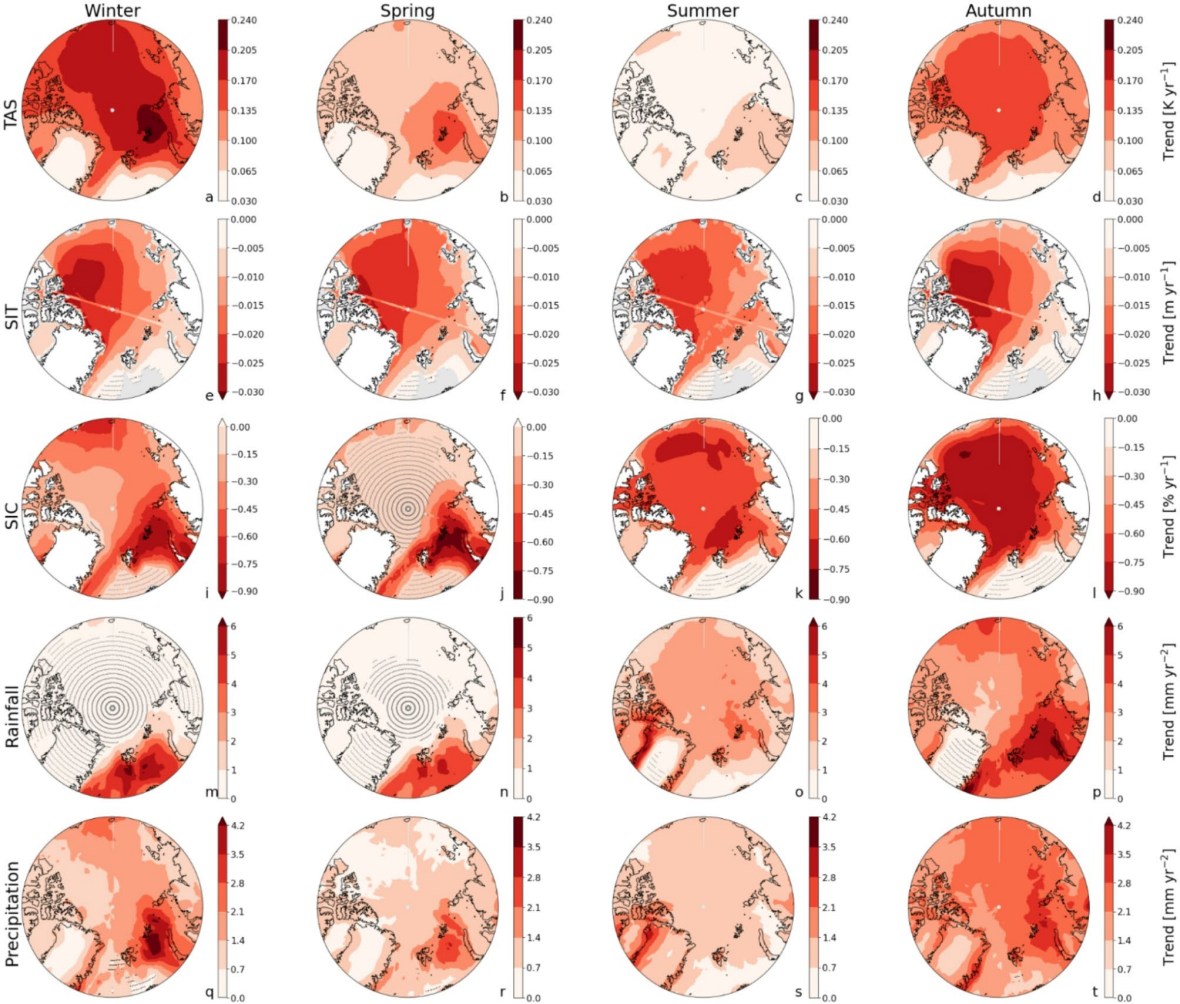
Geographical distribution of the trends in surface temperature (first row), sea ice thickness (second row), sea ice cover (third row), rainfall (fourth row), and total precipitation (fifth row). The values are calculated as the median linear trend of the CMIP6 models per location for winter (DJF, first column), spring (MAM, second column), summer (JJA, third column), autumn (SON, fourth column) over the period 1951–2100. Trends for SIT and SIC are negative, since sea ice is declining. Note the different colour scales per variable and season. White colours depict land without SIT and SIC. Stippled regions indicate non-significant trends (p-values > 0.05).

**Fig. 5:**
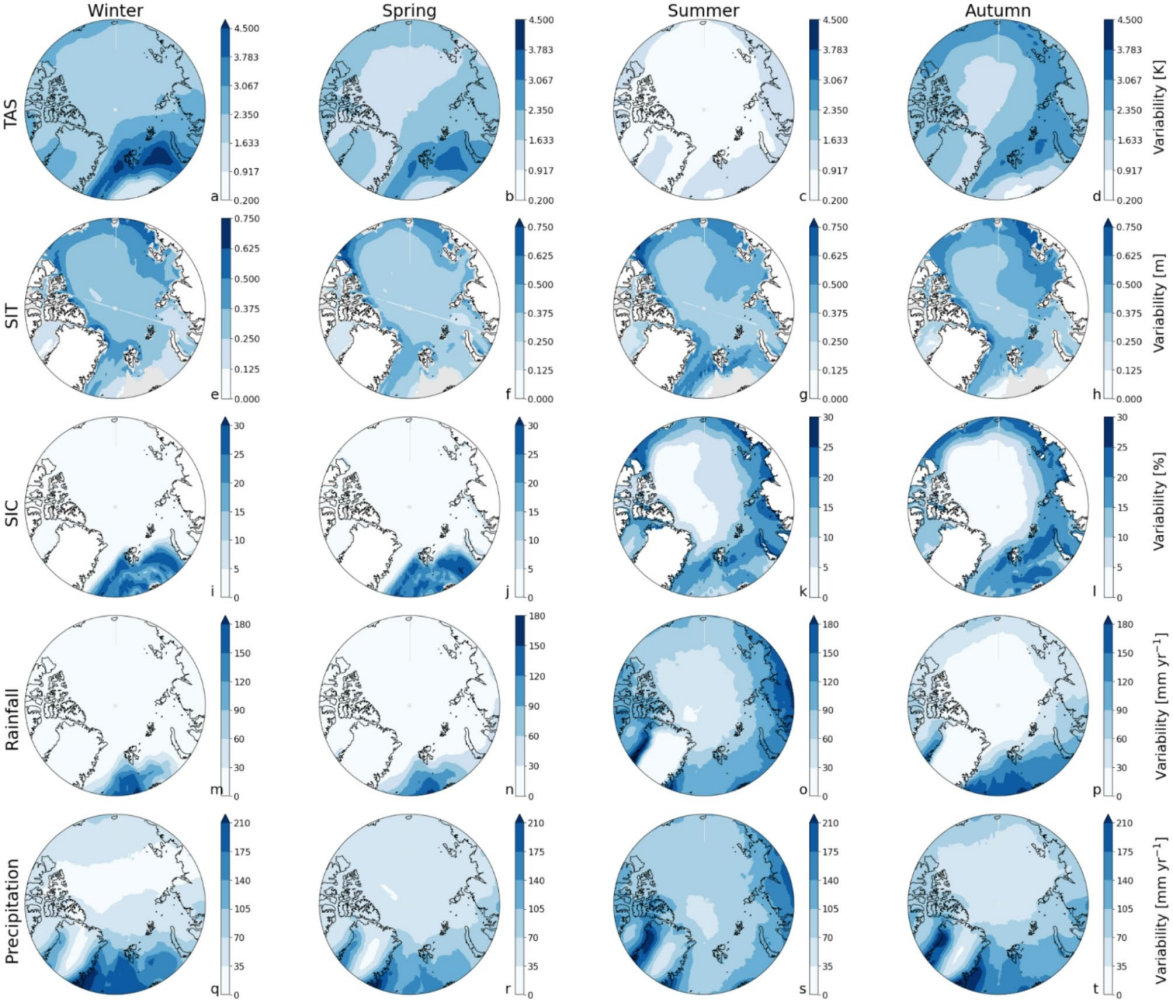
Geographical distribution of the variability for surface temperature (first row), sea ice thickness (second row), sea ice cover (third row), rainfall (fourth row), and total precipitation (fifth row). The values are calculated as the median variability (standard deviation of the detrended baseline period 1850–1949) of the CMIP6 models per location for winter (DJF, first column), spring (MAM, second column), summer (JJA, third column), autumn (SON, fourth column). Note the different colour scales per variable and season. White colours depict land without SIT and SIC.

Sea ice cover demonstrates greater resilience than sea ice thickness, particularly in regions dominated by multiyear thick ice (Fig. 3e-l), adding complexity to the timing of their transitions. In winter and spring, SIC transitions occur 17 and 31 years later than SIT, respectively (Fig. 2b, c), as reductions in ice thickness do not immediately lead to a decline in sea ice cover in these regions. In contrast, regions with currently relatively thin ice, e.g. the Barents Sea, exhibit SIT transitions that often lag behind SIC by up to 20 years (Fig. 3e-l), marking the final state before an ice-free state. This is especially true in regions where both variables are concurrently near the no-ice threshold. However, SIT exhibits greater ratio of variability and trend than SIC, especially in the Central Arctic (Figs. 4 and 5), introducing additional complexity to its spatiotemporal transition characteristics. This explains why, unlike SIT, SIC undergoes significant changes only when the ice is already thin, delaying its transition by up to 60 years compared to SIT—most notably in the Central Arctic during winter and spring (Fig. 3e-l). This lag is further exacerbated by the accelerated thinning of multiyear ice due to rising atmosphere and ocean temperatures^20^, which promote early SIT emergence.

SIT not only transitions earlier than SIC but also transitions almost simultaneously across all seasons. By summer 2050, only a few regions near the sea ice edge are expected to still be in the current SIT state, primarily due to the increased variability of sea ice around the edge, rather than the lack of a future thinning trend. In contrast, SIC exhibits distinct seasonal patterns due to the annual refreezing of sea water, which primarily results in thin, one-year ice. While SIT shows spatially-consistent reductions across seasons (Figs. 2 and 3), SIC transitions occur earliest in the Central Arctic during summer and autumn (Fig. 3k, l), with the latest transitions observed in winter and spring (Fig. 3i, j).

In summary, the transitions of TAS, SIC, and SIT across the Arctic show complex patterns that vary by season, region, and the current thickness of sea ice (Fig. 3a-l, Supplementary Information Fig. 4). While the earliest transitions are expected for autumn TAS (Fig. 2a), with SIT closely following in most regions (Figs. 2b and 3e-h), variability in sea ice conditions—such as thickness and seasonal refreezing—plays a crucial role in shaping these changes.

## Regional and seasonal variations in ToE – precipitation and rainfall

Similar to surface air temperature, precipitation and rainfall emerge first in autumn and winter (Fig. 2d and e m-t); in autumn, strong warming trends associated with sea ice loss^16^ considerably increase annual rainfall, particularly in the northern Barents and Kara Seas (Fig. 4p), where variability is also high, but trends dominate. In contrast, winter rainfall trends are moderate, but earlier emergences are facilitated by comparatively low variability. The exceptionally high variability in summer (> 80 mm year⁻¹) considerably delays rainfall emergences in almost all lower-latitude Arctic regions, where rainfall may not emerge before 2100 (Fig. 3o). Summer rainfall only emerges over the Greenland Ice Sheet (owing to a strong mean trend) and in the low-variability Central Arctic (Figs. 4o and 5o). Rainfall emerges earlier than precipitation in all seasons due to the accelerating effect of enhanced rainfall caused by Arctic warming^22^; since TAS emerges much earlier than precipitation (by on average 54 years; Fig. 2a and e), it follows that rainfall ToE – governed by trends and variability in precipitation as well as air temperature – will occur before that of precipitation. Independent of the season, total precipitation in the lower latitudes will not emerge, or only shortly, before the end of this century (Fig. 3q-t), especially over land where variability is high. Recent studies have shown that Arctic precipitation variability is strongly increasing due to increases in poleward moisture transport, especially in summer^19,23^, which further impedes the emergence of summer (and also spring) precipitation in any Arctic region before 2100 (Fig. 3s). The importance of evaluating grid-point metrics rather than Arctic averages is especially evident for precipitation and rainfall: the grid-point method (comBPnn) reveals significantly delayed emergences – from 16 up to 56 years later – compared to Arctic averages (Supplementary Information Fig. 1d, e), in which local variability is smoothed out by spatial averaging. Compared to an earlier study^16^ (based on 2 models), we find that rainfall ToE occurs later, even though the geographical pattern of ToE seems to be consistent, i.e. comparatively early over the Arctic Ocean, and relatively late over the adjacent continents. This difference likely arises from the limited number of models in [6] (since we show that model uncertainty is larger than member uncertainty; Supplementary Information Table 1) as well as possibly their use of rain days as a metric instead of precipitation and rainfall amounts. For example, the reported ToE in [6] for the duration of rain days emerges as early as 2042 in one model (2047 in the other model), which is a metric that is differently affected by rising temperatures than is the amount of rainfall. The reported emergences of the first and last rain days occurred 5–24 years later, but still earlier than any of the seasonal rainfall or precipitation ToEs reported in this study (with autumn and winter rainfall emerging first in 2077; Figs. 1 and 2).

## Discussion

Using a new, robust method and the most recent state-of-the-art global climate model simulations (CMIP6) to evaluate the time of emergence (ToE) of the climate trend in the Arctic region, we found that the emergence of all variables in most seasons and regions has not taken place yet, but will occur in the (near) future. We consistently allowed spatial variations in climate variability, which for sea ice cover/thickness result in a more representative – and much later than suggested in earlier studies –estimate of ToE. Supplementary Fig. 4 summarizes the main findings, showing that, generally, through its effect on sea ice cover, sea ice thickness exerts a dominant control over where and when ToE occurs with respect to the various variables. Generally, temperature and sea ice thickness are the first climate variables to emerge in specific Arctic regions^11–14,16^. Interestingly, sea ice thickness has already emerged in the Central Arctic due to its low temporal variability but strong thinning trend. Notably, Arctic sea ice cover emerges later than sea ice thickness, since sea ice cover changes mainly occur at the margins, whereas sea ice thinning can take place Arctic-wide. Precipitation and rainfall, despite exhibiting considerable trends, are found to emerge later in this century owing to strong, and increasing, natural variability.

Specifically, our multi-model, seasonal and spatially-varying analyses have resulted in three crucial findings. First, determining a distinct ToE for a large and diverse region such as the Arctic is associated with major uncertainties owing to huge spatial variations in local trends and variability. Simply area-averaging climate variables dismisses these regional characteristics and consequently results in unrepresentative estimates of ToE. While climate means and trends can either be first locally calculated and then averaged, or directly calculated based on regional averages, this is not applicable in case of temporal variability, for which it is important to capture changes from year to year locally, as regional averages allow for compensation of high and low anomalies, which unlike trends and means define the magnitude of variability. We show that the very variable Arctic sea ice cover emerges around 2020, and then only in the Barents and Kara Seas, while most other sea-ice covered regions emerge around 2030–2040 (summer and autumn) or 2060 or later (winter and spring) (Fig. 3e-h). These results are in strong contrast with existing sea ice related ToE estimates^16^, in which ‘Arctic sea ice’ is defined as the area-averaged SIE, and temperature-ToE are evaluated using Arctic averages, resulting in ToE-values of 2030–2045. Hence, calculating ToEs based on the spatially varying Arctic climate shifts ToE later by decades.

Second, climate model-based ToE should be evaluated from a large set of models, as intermodel differences in ToE for all climate variables are substantial (Fig. 3). In addition, there is also considerable spread across model members, but this is smaller than the intermodel spread (Supplementary Information Table 1), suggesting that sampling model variability using an multi-model ensemble is crucial^13,15^.

Third, our results reveal that seasonal and regional variability in ToE is substantial, suggesting that in order to define at what point in time a new Arctic climate emerges, it is advisable to select representative months and study specific regions to get a more meaningful (i.e. less scattered) estimate of ToE. We find that for most seasons, temperature and sea ice thickness emerge first in the Central Arctic, whereas sea ice cover and rainfall emerge first in the Barents Sea region.

Overall, our relatively late ToE-values might be surprising considering the fact that Arctic climate trends are generally larger than in other regions of the world. This demonstrates that very strong natural variability (interannual, decadal) in the Arctic plays an important role in “delaying” ToE. We suggest that instead of evaluating the emergence of Arctic-wide annual averages, the focus should be on finer regional and seasonal scales, where indications of recent past or near-future emergence is still a clear and alarming indicator of the Arctic climate transforming into a new, previously unknown state.

Evaluating an accurate value of ToE is particularly important for ecosystems because these need to adapt to a new climate state when climate conditions change beyond the current envelope to values that were never experienced before^24,25^. The asynchronous emergence of different variables poses additional challenges, as flora and fauna often depend crucially on specific combinations of climate variables (e.g. temperature and precipitation/rainfall/snowfall for Arctic birds)^26^. Likewise, there are severe consequences for indigenous communities in the Arctic, which will be strongly impacted by infrastructural problems due to permafrost melt, or changes to the ocean ecosystem which alter Arctic fishing practices^27–31^. Our findings can thus be used as a guide for adaptation strategies in a rapidly changing Arctic^32^ in which the emergence of a new climate state exhibits strong geographical and seasonal variations, and differs crucially between the various climate variables.

## Methods

### Climate model data

In all analyses we used 15 Coupled Model Intercomparison Project, phase 6 (CMIP6) state-of-the-art global climate models (Supplementary Information Table 2). These models simulated climate change using a set of emission scenarios describing different development paths for the period 2015–2100. Here we primarily use the upper boundary of the range of these scenarios (SSP5-8.5), for which the combined greenhouse, aerosol and other radiative forcings in the year 2100 totals 8.5 W m^− 2^ (we additionally used other scenarios to assess scenario-uncertainty, see Supplementary Information Table 1). We merged these with their respective CMIP6 historical simulations for the period 1850–2014, resulting in 15 models with 250 years (1850–2100) of monthly climate model output. We use all models for which data coverage was complete; one ensemble member per model (the first) was used (for CanESM5 we additionally used 50 members to assess the multi-member uncertainty). All data were regridded to a spatial resolution of 1º in longitude and 1º in latitude, and resampled to a seasonal or annual temporal resolution. In this study we define the Arctic as the region between 70–90°N because most of the climate changes (e.g. precipitation and sea ice) occur in or near the Arctic Ocean, north of 70°N^8^. Finally, we validate these models by comparing the Arctic mean value of each variable (median of all models) to the Arctic mean value of ERA5 (except for SIT, which is not available in ERA5), for the period 1980–2010 (Supplementary Information Table 3). This revealed the well-documented cold bias of CMIP6 models^23,33,34^, which is most pronounced in winter, with mean temperatures approximately 3 °C lower than ERA5. However, it is important to note that ERA5 is not a perfect reference for Arctic climate, and CMIP6 models are often closer to observations^33^. Other studies have also highlighted considerable biases in Arctic precipitation and the surface energy budget over sea ice in ERA5^35,36^. Therefore, we do not expect the 15 models to perfectly align with ERA5. Regarding precipitation, the models are between 4 and 15% drier than ERA5 depending on the season, while differences in SIC and rainfall vary across seasons: rainfall is slightly higher than ERA5 in all seasons except summer, and SIC values are very comparable to ERA5 in winter and spring, but 10–11% lower in summer and autumn (Supplementary Information Table 3).

## Calculation of ToE

In this study we split the time series into two distinct parts by assigning a constant in time and common (for all grid cells, variables and models) breakpoint at year 1950 (similar [16]). This posits that the time series is composed of two distinct parts: the 100-year long “baseline period” (1850–1949), and the 150-year long “new state” (1950–2100). The Time of Emergence (ToE) is defined using the 95% confidence interval (CI) of the baseline variability, as calculated from the variable’s linearly detrended baseline period, and stays outside of the CI for at least 10 consecutive years. This is achieved by fitting a rolling window of 10 years in steps of one year, and counting the number of years outside the 95% CI. As soon as a rolling window satisfies this condition (count = 10), its last year (i.e. the 10th consecutive year outside the 95% CI) is defined as the ToE (Supplementary Information Fig. 5). We use a 10-year rolling window because it effectively smooths interannual variability while preserving decadal-scale trends, providing a stable yet responsive measure of long-term change^12,16,37^. We performed sensitivity tests instead using five and seven years for the window period, which only resulted in minor changes to the resulting ToE values. Falling outside of the 95% CI, for TAS, rainfall and precipitation means surpassing the 97.5th percentile, whereas for SIC and SIT it means dropping below the 2.5th percentile of the variable’s linearly detrended baseline period.

## Variability and trend

ToE is governed by both the variability of the baseline period (from 1850 until 1949) and the trend of the new state (from 1950 until 2100), in the sense that noisier baseline periods (wider 95% CI) and/or weaker trend (smaller slopes in the new state) tend to delay ToE (Supplementary Information Fig. 6). Here, variability is defined as the standard deviation of the linearly detrended baseline period, and trend as the slope of the linear fit of the new state (Supplementary Information Fig. 5c).

### Grid point calculations per model

All calculations are first performed per model, after which the median of all models provides the final value of ToE. We use three different levels of spatial resolution: Arctic mean, five subregions and per grid point. In the first case, calculations are performed on the spatially weighted mean of the Arctic (see Fig. 1), while in the second case this is done on the weighted mean of each of five subregions in the Arctic (see Supplementary Information Figs. 1 and 2). Finally, in the third case all calculations are done per model grid point to represent spatial variability as accurately as possible (comBP). For each model, grid points failing to produce a ToE-value during the 21st century (ToE ≤ 2100) are assigned a ToE-value of 2101 (comBPnn, which is the reference method throughout the paper). At these locations, the new state will emerge only after 2100, or possibly never. As a result, actual Arctic mean ToE-values for especially precipitation and rainfall may thus sometimes be later than reported in this study, because any ToE occurring after the end of the 21st century is assigned a value of 2101. Supplementary Information Fig. 8 shows the number of models that produce a ToE-value before 2100 for all variables, seasons, and locations.

### Estimating and comparing various sources of uncertainty

In this study we employed multiple ToE calculation methods (Arctic mean, five subregions, comBP, comBPnn) and various CMIP6 models (Supplementary Information Fig. 7). Moreover, we tested the sensitivity of our results to various future emission scenarios (SSP-1.26, SSP-2.45, SSP-5.85) and internal variability (using 50 members from the model CanESM5). The three latter sources of uncertainty (models, scenarios and model members) were quantified by calculating the per-grid point standard deviation of ToE (e.g. the standard deviation of 15 ToE values from 15 models) and then spatially averaging ToE over the entire Arctic. The method uncertainty was calculated as the standard deviation of four values for the degree of spatial averaging: Arctic mean, five subregions, comBP, comBPnn, for the entire Arctic. This analysis shows that the model uncertainty (Supplementary Information Table 1) clearly dominates for all variables and seasons, illustrating the importance of using as many models as possible. We also explicitly quantified the difference in ToE between scenarios SSP-5.85 and SSP-1.26, as this is useful information with regards to mitigation policies (Supplementary Information Table 4). For most variables and seasons, the ToE for SSP-5.85 occurs earlier (by up to 22 years in some cases) than for SSP-1.26 because of the comparatively large climate trends in SSP-5.85.

## Electronic supplementary material

Below is the link to the electronic supplementary material.

Supplementary Material 1

## Data Availability

CMIP6 model output is freely available through a distributed data archive developed and operated by the Earth System Grid Federation (ESGF). Data sets generated during the current study are available from the corresponding author on reasonable request. All requests for materials and correspondence should be directed to R.B. (r.bintanja@rug.nl).

## Code availability

Any code and scripts used to analyse the climate model data are available from the corresponding author on reasonable request. All requests for materials and correspondence should be directed to R.B. (r.bintanja@rug.nl).
