# Supplementary material for: The time of emergence of Arctic warming, wetting and sea ice melting
Source: Sci Rep. 2025 Apr 12;15:12626. doi: 10.1038/s41598-025-96607-1 (PMC11993599; doi:10.1038/s41598-025-96607-1)
Supplement: Supplementary file 1 — Supplementary Material 1 [file 41598_2025_96607_MOESM1_ESM.docx]

### **Supplementary Information**


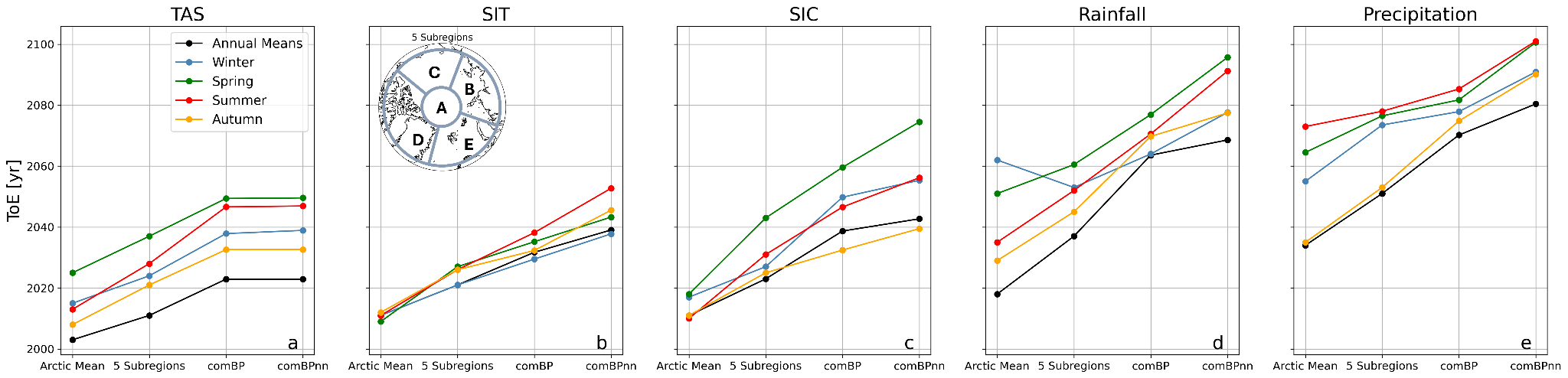


Supplementary Information Figure 1. Arctic mean ToE of a) TAS, b) SIT, c) SIC, d) rainfall and e) total precipitation, per season/annual (line colours) and method (x-axis, in order of increasing spatial resolution). Four methods were used in the calculation of Arctic mean ToE, “Arctic Mean”: ToE was calculated on the Arctic mean of each variable, “5 subregions”: ToE calculations were performed on the weighted means of 5 subregions (Central Arctic - A, East Siberia - B, Chukchi Sea - C, Greenland and Canada - D, Barents Sea - E; see figure insert) and then averaged over the Arctic, “comBP”: ToE calculations were performed per grid point, with “comBPnn” equaling “comBP” but with grid points failing to produce a ToE during the 21st century being assigned a ToE-value of 2101, after which the Arctic mean was calculated.


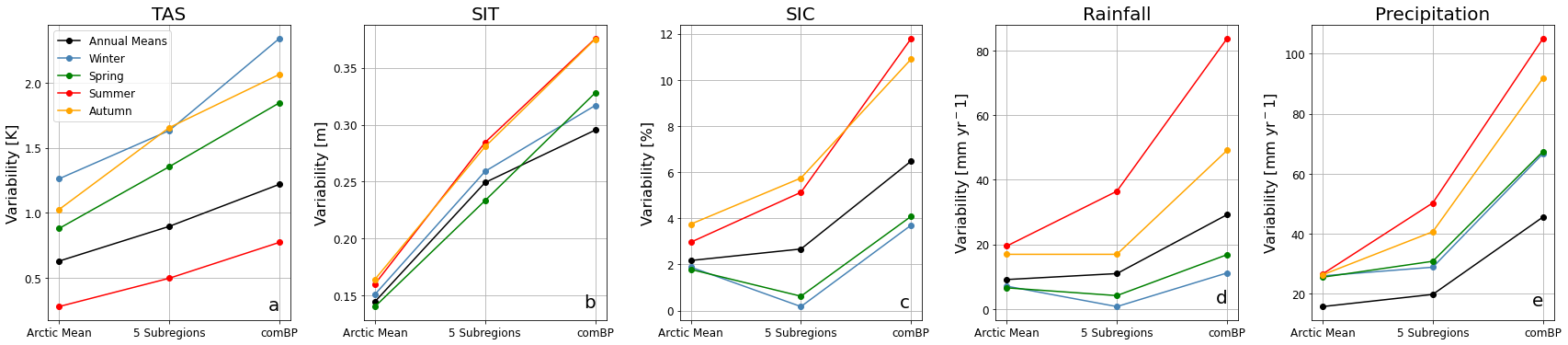


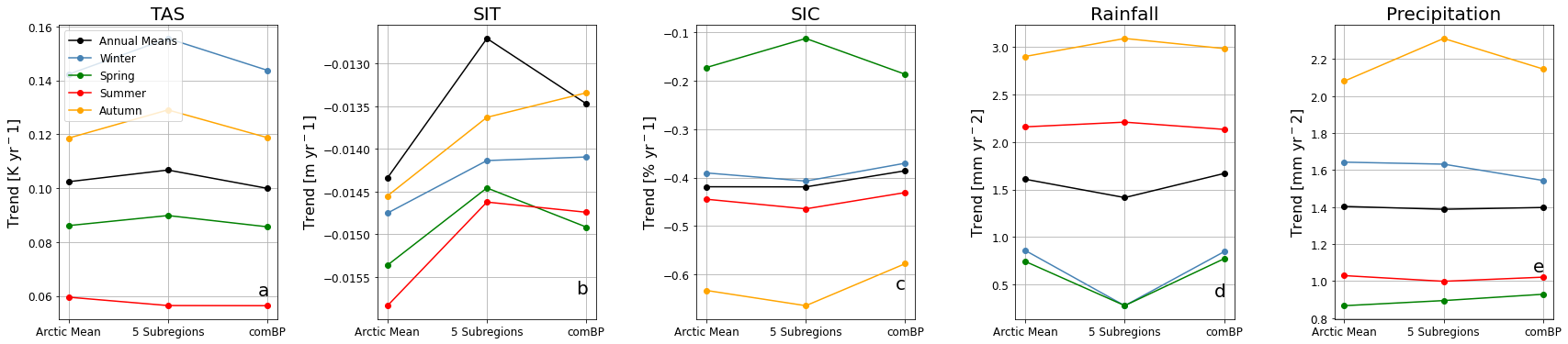


Supplementary Information Figure 2. Arctic mean variability and trend of a) TAS, b) SIT, c) SIC, d) rainfall and e) total precipitation, per season (line colours) and method (x-axis, in order of increasing spatial resolution). Four methods were used in the calculation of the ratio, “Arctic Mean”: signal and variability were calculated on the Arctic mean of each variable, “5 subregions”: signal and variability calculations were performed on the weighted means of five subregions (see caption and insert of Supplementary Figure 1) and then averaged, “comBP”: signal and variability calculations were performed per grid point, after which the Arctic mean was calculated. Outliers occur for regions/variables/methods with very low temporal variability.


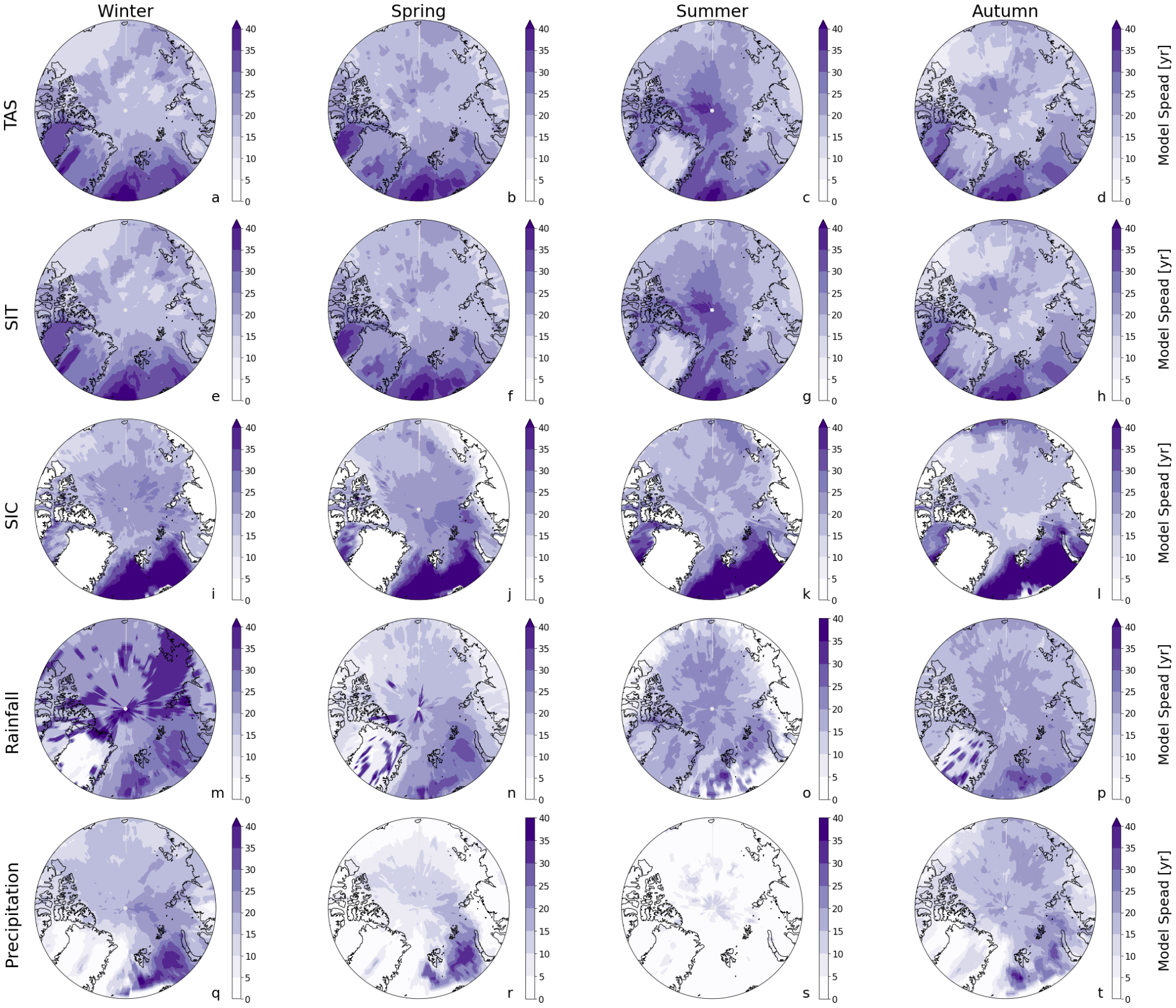


Supplementary Information Figure 3. Geographical distributions of intermodel spread in ToE for surface air temperature (first row), sea ice thickness (second row), sea ice cover (third row), rainfall (fourth row), and total precipitation (fifth row). Intermodel spread is evaluated as the per-grid point standard deviation of ToE estimates from all 15 CMIP6 models, for winter (DJF, first column), spring (MAM, second column), summer (JJA, third column), and autumn (SON, fourth column). White colour depicts land without SIT and SIC.


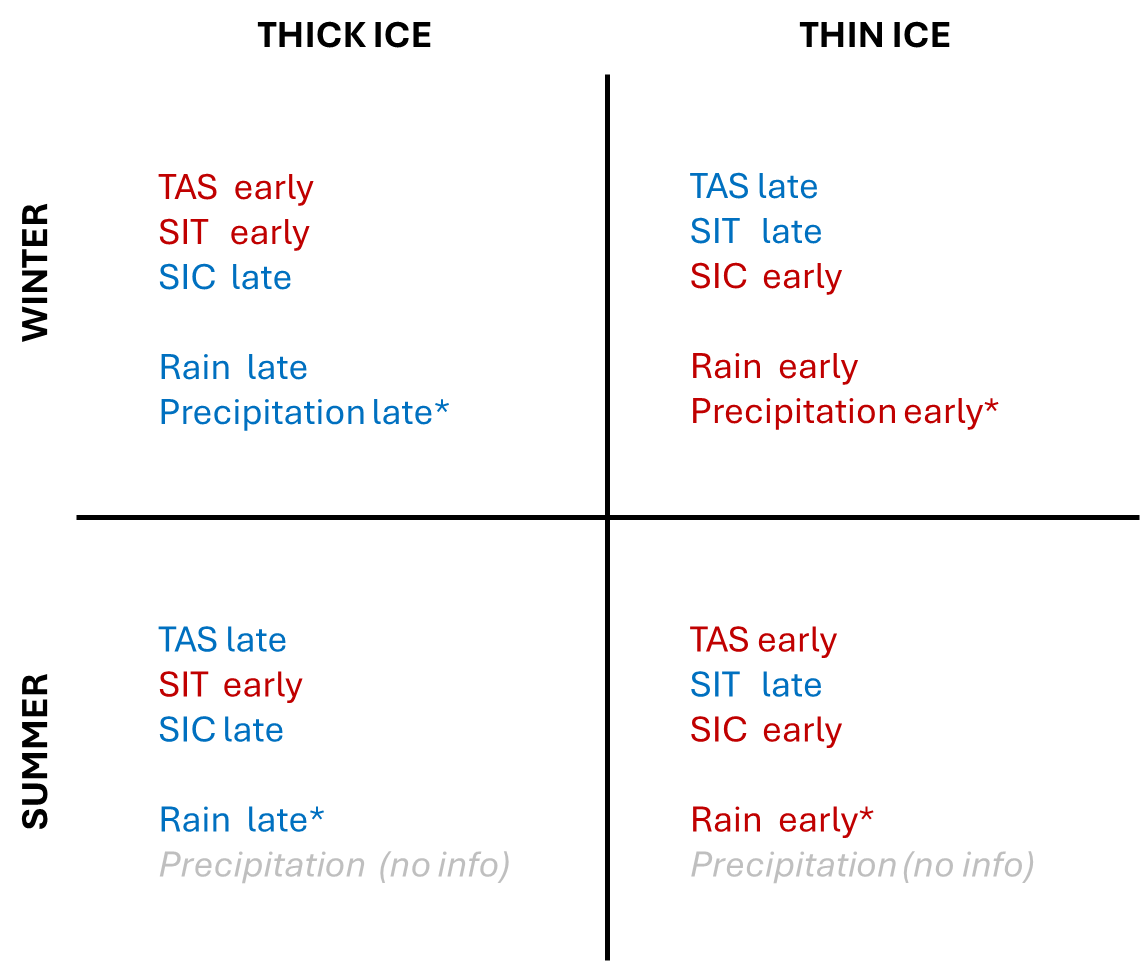


Supplementary Information Figure 4. Schematic diagram illustrating the characteristics of ToE for different variables across relatively thick (multiyear) and thin (one-year) sea ice regions (two columns) and two seasons (winter and summer, two rows). The terms “early” (red) and “late” (blue) indicate the ToE of a specific variable within a given season and are used to compare thick (left column) versus thin (right column) sea ice regions. The general patterns are derived from Figure 3. Most variables exhibit consistent spatial behavior between winter and summer, meaning that if ToEs are relatively early (or late) in thick ice regions during one season, they generally follow the same pattern in the other. The exception is TAS, where this pattern reverses: in thick ice regions, ToEs occur earlier in winter but later in summer compared to thin ice regions. Variables with an asterisk (*) indicate cases where fewer than 50% of models produced ToEs over large parts of the Arctic (precipitation in winter and rainfall in summer). Variable names in grey indicate cases where fewer than 50% of models produced ToEs across the entire Arctic (precipitation in summer).


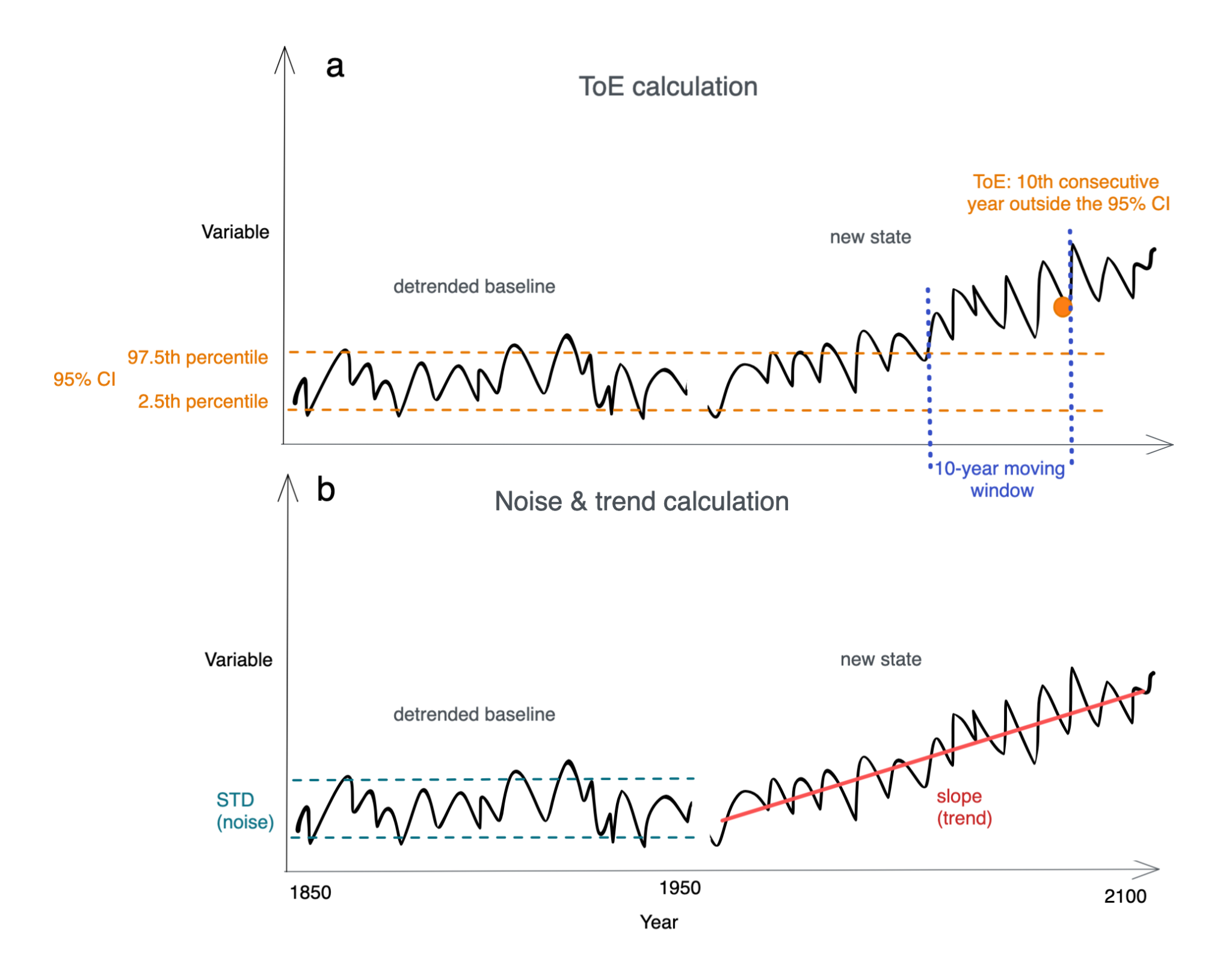


Supplementary Information Figure 5. Schematic diagrams describing the basic principles behind the ToE, variability and trend calculations: a) the 95% confidence interval (CI) is calculated using the baseline and the 10th consecutive year outside of the 95% CI (yellow dot) is the ToE, and b) variability and trend as calculated using data from the baseline and the new state, respectively.


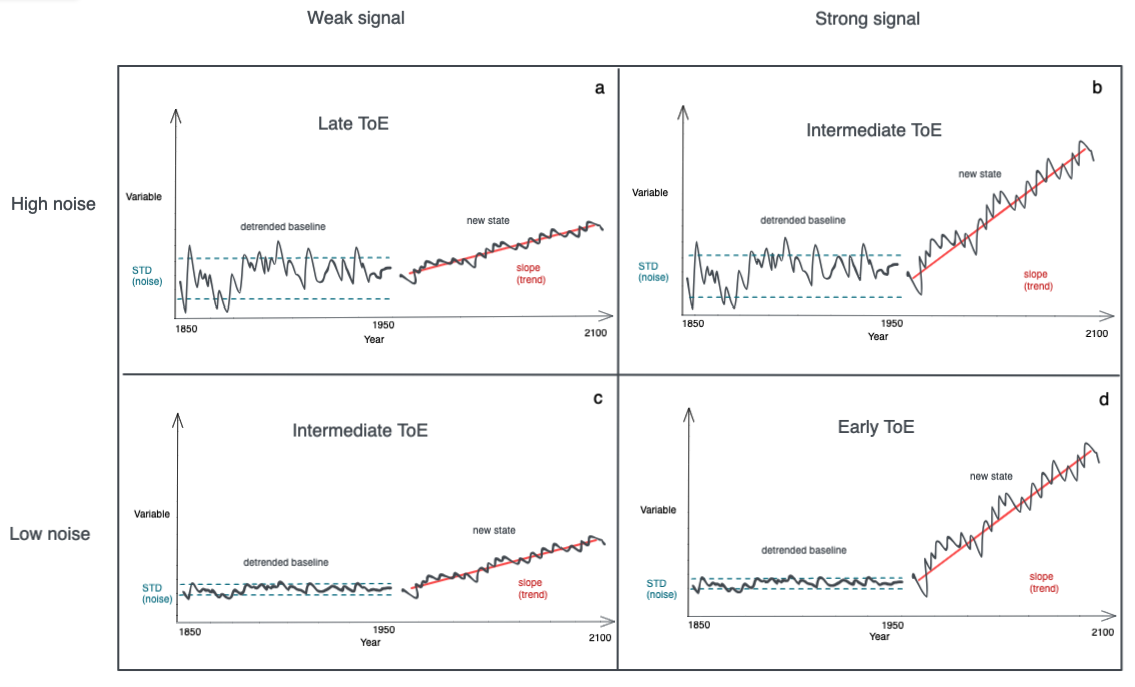


Supplementary Information Figure 6. Schematic diagrams depicting four different theoretical cases. In case a) the “noisy” baseline period causes a large uncertainty range, and in combination with a weak trend (small slope) results in a late ToE (late in the 21st century or even thereafter). In b) the “noisy” baseline period is combined with a strong trend during the new state, and results in intermediate ToE values, something happening also in case c) where the low baseline variability is combined with a weak new state trend. Finally, in case d) the strong new state trend surpasses the small uncertainty range (low-variability baseline period) quickly, which yields an early ToE.


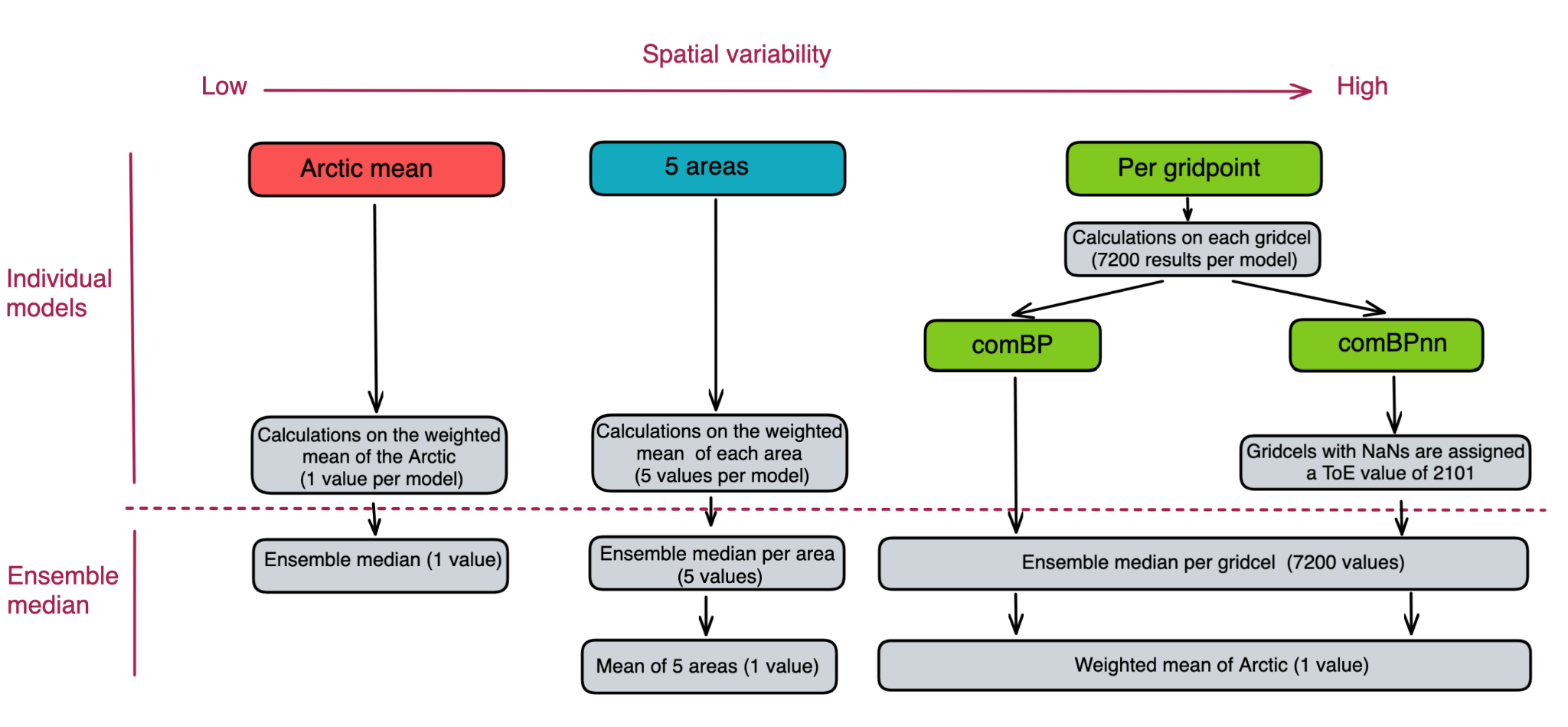


Supplementary Information Figure 7. Schematic representation of the data processing and calculation methods used in this study. Note that in evaluating model-mean ToE values, first the ToE of each model was calculated, after which these were averaged.


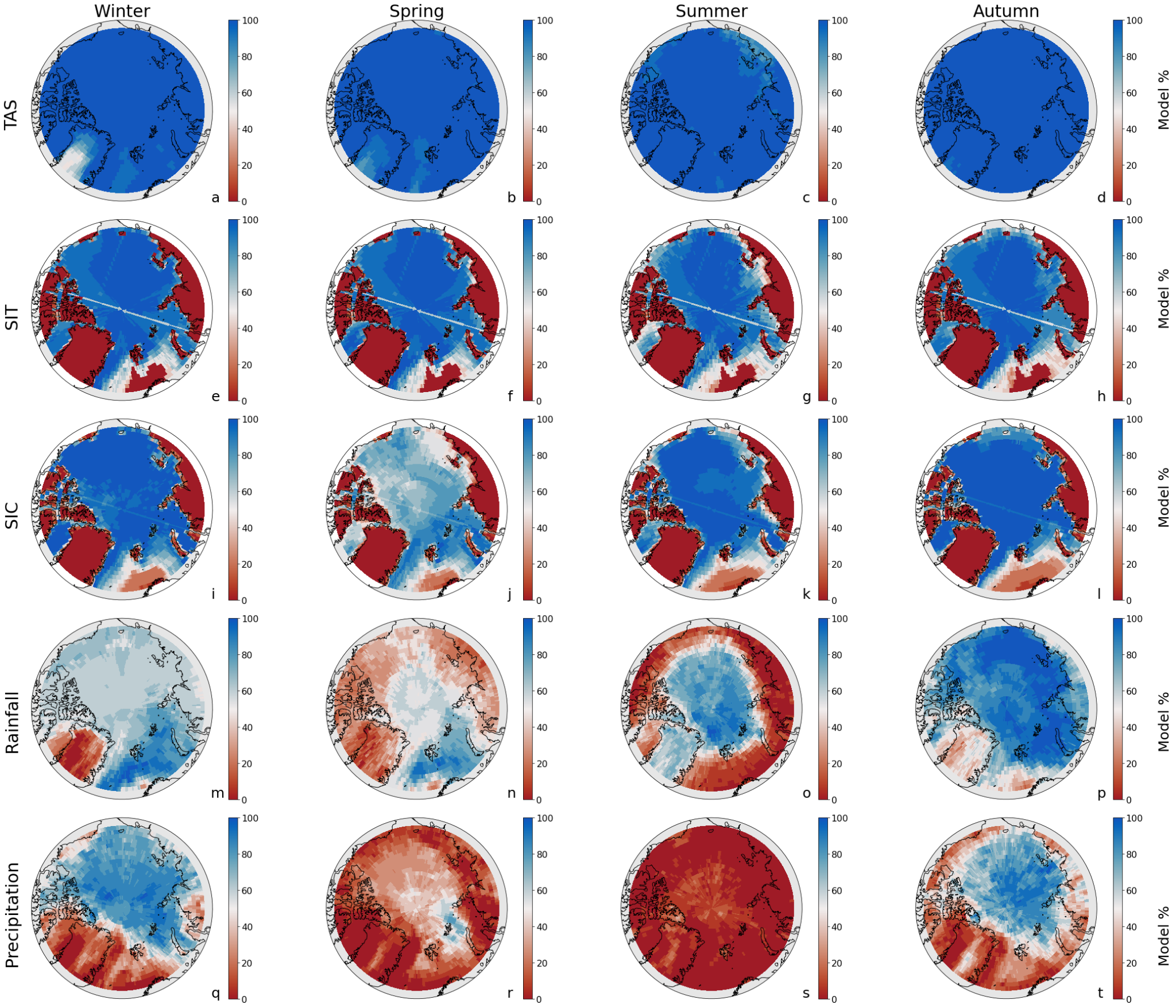


Supplementary Information Figure 8. Geographical distributions of percentage of models that produce a ToE within the 21st century, using our per grid point method (comBP), for surface air temperature (first row), sea ice thickness (second row), sea ice cover (third row), rainfall (fourth row), and total precipitation (fifth row). Calculations represent all 15 CMIP6 models, for winter (DJF, first column), spring (MAM, second column), summer (JJA, third column), and autumn (SON, fourth column).


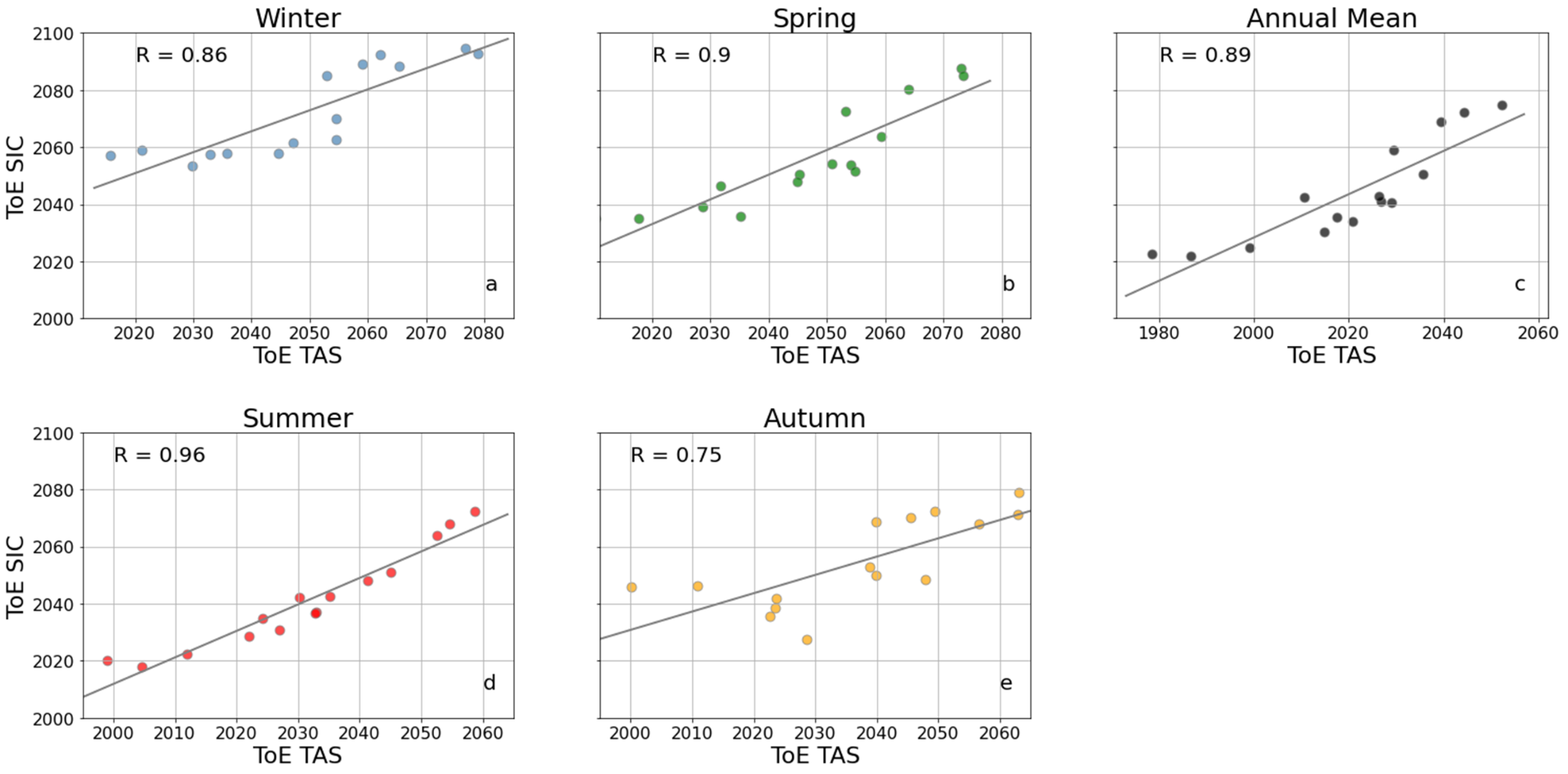
Supplementary Information Figure 9. Scatter plots showing the relationship between TAS (x-axis) and SIC (y-axis) ToEs for all 15 models used in this study. ToEs are calculated using seasonal and annual means for: a) winter, b) spring, c) annual, d) summer, and e) autumn. Each model is represented as a dot, with a linear regression line overlaid and the correlation coefficient (R) displayed in each subplot.

|  | **TAS** | **SIT** | **SIC** | **RAIN** | **PREC** |  |
| --- | --- | --- | --- | --- | --- | --- |
| **MMe** | 3 | 0 | 0 | 2 | 2 | **Annual Means** |
| **MMo** | 20 | 18 | 17 | 15 | 13 |  |
| **Scenario** | 3 | 2 | 7 | 11 | 10 |  |
| **Method** | 11 | 9 | 11 | 7 | 11 |  |
| **MMe** | 3 | 0 | 0 | 2 | 2 | **Winter** |
| **MMo** | 18 | 18 | 15 | 17 | 10 |  |
| **Scenario** | 6 | 3 | 9 | 10 | 5 |  |
| **Method** | 11 | 8 | 11 | 13 | 10 |  |
| **MMe** | 3 | 0 | 0 | 1 | 1 | **Spring** |
| **MMo** | 18 | 18 | 16 | 14 | 4 |  |
| **Scenario** | 11 | 7 | 11 | 2 | 0 |  |
| **Method** | 13 | 8 | 12 | 10 | 9 |  |
| **MMe** | 2 | 0 | 0 | 2 | 0 | **Summer** |
| **MMo** | 18 | 19 | 17 | 8 | 1 |  |
| **Scenario** | 12 | 6 | 9 | 5 | 0 |  |
| **Method** | 7 | 7 | 12 | 5 | 11 |  |
| **MMe** | 2 | 0 | 0 | 1 | 2 | **Autumn** |
| **MMo** | 17 | 19 | 16 | 14 | 9 |  |
| **Scenario** | 5 | 5 | 6 | 9 | 6 |  |
| **Method** | 9 | 8 | 9 | 7 | 9 |  |

Supplementary Information Table 1. Inter-member (MMe), inter-model (MMo), scenario (Scenario) and method (Method) uncertainty of ToE estimates (in years), for all five variables (columns). For the first three sources of uncertainty, calculations were performed per grid point (e.g. standard deviation of 15 ToE values from 15 models) and finally spatially averaged over the entire Arctic. See Methods for further details.

|  | **CMIP6 model** | **Lat x Lon** | **Vertical Levels** | **Top Pressure Level** |
| --- | --- | --- | --- | --- |
| 1 | ACCESS-CM2 | 1.250^o^ x 1.875^o^ | 85 | 85 *km* |
| 2 | BCC-CSM2-MR | 1.125^o^ x 1.120^o^ | 46 | 1.46 *hPa* |
| 3 | CanESM5-CanOE | 2.8^o^ x 2.8^o^ | 49 | 1 *hPa* |
| 4 | CanESM5 | 2.8^o^ x 2.8^o^ | 49 | 1 *hPa* |
| 5 | CNRM-CM6-1 | 1.4^o^ x 1.4^o^ | 91 | 78.4 *km* |
| 6 | CNRM-ESM2-1 | 1.24^o^ x 1.24^o^ | 91 | 78.4 *km* |
| 7 | EC-Earth3-Veg | 0.7^o^ x 0.7^o^ | 91 | 0.01 *hPa* |
| 8 | EC-Earth3 | 0.7^o^ x 0.7^o^ | 91 | 0.01 *hPa* |
| 9 | GFDL-ESM4 | 1.00^o^ x 1.25^o^ | 49 | 1 *hPa* |
| 10 | IPSL-CM6A-LR | 1.26^o^ x 2.50^o^ | 79 | 80 *km* |
| 11 | MIROC-ES2L | 2.8^o^ x 2.8^o^ | 40 | 3 *hPa* |
| 12 | MIROC6 | 1.4^o^ x 1.4^o^ | 81 | 0.004 *hPa* |
| 13 | MPI-ESM1-2-LR | 0.9375^o^ x 0.9375^o^ | 95 | 0.01 *hPa* |
| 14 | MRI-ESM2-0 | 1.125^o^ x 1.125 | 80 | 0.01 *hPa* |
| 15 | UKESM1-0-LL | 1.250^o^ x 1.875^o^ | 85 | 85 *hPa* |

Supplementary Information Table 2. Horizontal resolution (lat, lon), vertical resolution (number of levels), and top pressure level of the 15 CMIP6 models used in this study.

|  | **TAS (K)** | **SIC (% cover)** | **Rainfall (mm/year)** | **Precipitation (mm/year)** |
| --- | --- | --- | --- | --- |
| Annual mean | 1.4 | 2.6 (3.2) | 7.7 (8.2) | 23.8 (7.0) |
| Winter | 3.0 | -0.8 (-0.9) | -0.7 (-4.1) | 42.2 (15.1) |
| Spring | 1.4 | 0.2 (0.2) | -0.6 (-3.0) | 23.6 (9.2) |
| Summer | 0.8 | 7.8 (10.8) | 25.2 (10.0) | 18.8 (4.3) |
| Autumn | 0.4 | 8.1 (10.4) | -3.7 (-4.4) | 41.8 (10.4) |

Supplementary Information Table 3. Comparison of model mean Arctic mean values with ERA5. Differences are shown as absolute values, representing the Arctic mean value of ERA5 minus the median Arctic mean value of the CMIP6 models, for the period 1980 – 2010. Values in brackets indicate relative differences (in %).

|  | **TAS** | **SIT** | **SIC** | **Rainfall** | **Precipitation** |
| --- | --- | --- | --- | --- | --- |
| Annual mean | -5.4 | -7.7 | -13.6 | -21.8 | -19.4 |
| Winter | -10.0 | -6.0 | -24.2 | -19.7 | -9.5 |
| Spring | -22.3 | -12.3 | -19.6 | -3.3 | * |
| Summer | -22.1 | -10.9 | -21.0 | -9.4 | * |
| Autumn | -6.6 | -4.1 | -10.7 | -17.9 | -10.8 |

Supplementary Information Table 4. Difference in ToE between scenarios SSP-5.85 and SSP-1.26. A negative number means an earlier ToE in the strongest scenario (SSP-5.85). For most variables and seasons, the ToE for SSP-5.85 occurs before that of SSP-1.26 because of the larger trends in SSP-5.85. *Not enough data to determine the difference.
